# Death of a Hospital Founder: the late Mr. George Hanbury

**Published:** 1912-12-14

**Authors:** 


### Death of a Hospital Founder.

### THE LATE MR. GEORGE HANBURY.

On Saturday, November 30, the death took place of Mr. George Hanbury, the beloved and honoured treasurer and a founder of the Paddington Green Children's Hospital, at the great age of eighty-three.

Among the numerous philanthropic societies and charitable institutions in which the late Mr. Hanbury took an interest, it may confidently be stated that the Children's Hospital on Paddington Green (with its Convalescent Home at "Fair View," Slough) was the chief. In November 1881, when it was proposed by the committee of the North-West London Free Dispensary for Sick Children in Bell Street, Edgware Road, to enlarge the dispensary into a cottage hospital, Mr. George Hanbury was invited to become, and was duly elected, chairman and treasurer, and was mainly responsible for securing the funds required for the new undertaking. The hospital was established on its present freehold site in August 1883 with twenty-three beds. It was rebuilt and enlarged with forty-six beds in 1895 at a cost of nearly £20,000. In 1904, owing to the state of his health, Mr. Hanbury resigned the chairmanship, which was taken up by the present chairman, Mr. Douglas Owen. Mr. Hanbury, however, remained treasurer, despite his ill-health, until his death, and frequently attended the meetings of the committee.

In 1911 the out-patient department was rebuilt and extended at a cost of £13,000, and was opened, as The Hospital on November 25 recorded at length, by H.R.H. Princess Louise, Duchess of Argyll. In all this work Mr. George Hanbury spared himself neither time nor trouble, and it is a matter of great gratification to the committee and supporters of the institution (as it undoubtedly was to himself) that he lived to see the highly successful consummation of his cherished hope for the ultimate establishment of a well-built and properly equipped hospital for children in this district of London, himself the founder. Mr. Hanbury's care and solicitude for the welfare of helpless and sick children of the poor was a marked trait in his noble character, and for many years his regular visits to the wards of the hospital on Sunday afternoons was a happy custom not only to himself but to the little patients. His gentle disposition and uniform consideration endeared him to all those with whom he came in contact at the hospital. His many and most generous contributions to the hospital funds, often greatly needed, are gratefully remembered by the committee of management, whilst to the warm and practical interest he bestowed in its general affairs the hospital owes much of its successful progress. The last of his numerous gifts to the hospital was a tile picture panel for the wall of the new out-patient waiting hall.

The funeral took place at Burnham, Maidenhead, on Wednesday last week, when those present included the following representatives from the Children's Hospital, Paddington Green, viz. : Mr. Douglas Owen, chairman of the hospital; Mr. J. Balfour Allan; Mr. L. C. Wakefield; Mr. Charles Marsham; Dr. L. G. Guthrie, senior physician to the hospital; Mr. W. H. Pearce, secretary; Miss Anderson, a former matron of the hospital; Miss E. Sheriff Macgregor, matron of the hospital; and Miss Archibald, matron of the convalescent home.

